# Response to Reader Feedback and Comments on Our Published Paper “Evaluating the Preparedness of Healthcare Providers for Prone Position CPR Across Jordan's Healthcare Sectors”

**DOI:** 10.1002/hsr2.71217

**Published:** 2025-09-07

**Authors:** Ahmad Al Hroub, Sami Al‐Yatim, Majeda Al‐Ruzzieh

**Affiliations:** ^1^ King Hussein Cancer Center Amman Jordan

Dear Editor,

Thank you for sharing the reader's valuable feedback on our recent article, “Evaluating the Preparedness of Healthcare Providers for Prone Position CPR Across Jordan's Healthcare Sectors.” We appreciate the opportunity to offer clarification. Most of the points raised were acknowledged in the limitations and recommendations sections of our manuscript [1]. We agree that these factors impact the generalizability of our findings, and we have suggested future research using objective evaluations, broader samples, and longitudinal methods to address them.

We also recognize additional potential confounding variables such as shift timing, clinical experience, and institutional policies. While not assessed in this study, we acknowledge their importance and recommend their inclusion in future work.

## 1 Response to Reader's Comments

- *Comment:* The sole use of self‐evaluation allows room for subjectivity and will not necessarily represent true clinical proficiency; external simulation‐based assessments have been demonstrated to be more accurate in quantifying readiness.
- *Response:* We agree that relying solely on self‐evaluation may limit the generalizability of our findings. This issue was addressed in the recommendations section of our published paper, where we suggest future studies should include objective, simulation‐based assessments.
- *Comment:* The study does not assess the quality, content, or frequency of previous training—variables consistently shown to have a deep effect on resuscitation performance. Without this, one cannot know if increased scores on the preparedness instrument reflect good training or merely pre‐exposure.
- *Response:* We acknowledge that our study did not evaluate the quality, content, or frequency of prior training, all of which are important factors affecting preparedness. This limitation was noted in our paper, and we have recommended that future research incorporate detailed, objective assessments of these training variables.
- *Comment:* All participating hospitals were urban, tertiary‐level hospitals, which limits generalizability to rural or lower‐resource settings, where preparedness may be even lower. In these environments, there may be less access to formal training, standardized orders, or simulation labs, and again, the preparedness gap widens. Extending the hospital sample to include a greater diversity of hospitals may help increase the external validity of the results.
- *Response:* We agree that focusing solely on urban, tertiary‐level hospitals restricts the generalizability of our findings. This limitation is acknowledged in our paper, and we recommend future research include hospitals from a broader range of settings to better reflect preparedness in diverse environments.
- *Comment:* Shift time, past clinical experience with PPCPR, and institutional policy were not controlled for; each of these may be confounding factors that can influence levels of preparedness. Variability in exposure, workload, and protocol awareness between shifts and institutions may result in variable training outcomes.
- *Response:* We recognize that shift timing and prior clinical experience were not controlled for and may serve as confounding factors. Although not addressed in our current paper, we consider this an important area for future research.
- *Comment:* The cross‐sectional design only captures a snapshot in time, and longitudinal follow‐up would be required to understand skill retention and the effect of training. Short of following through over time, it is hard to measure whether gains in preparedness are lasting or temporary effects.
- *Response:* We acknowledge that the cross‐sectional nature of our study limits our ability to assess skill retention or long‐term training outcomes. This limitation was noted in our paper, and we recommend future longitudinal studies to explore the sustainability of preparedness improvements.

We sincerely appreciate this constructive feedback, which aligns with and reinforces the direction we have proposed for further investigation into PPCPR preparedness.

## Author Contributions

**Ahmad Al Hroub:** writing – original draft. **Sami Al‐Yatim:** writing – review and editing. **Majeda Al‐Ruzzieh:** writing – review and editing.

## Ethics Statement

The authors have nothing to report.

## Consent

The authors have nothing to report.

## Transparency Statement

The corresponding author Ahmad Al Hroub affirms that this manuscript is an honest, accurate, and transparent account of the study being reported; that no important aspects of the study have been omitted; and that any discrepancies from the study as planned have been explained.

## Data Availability

Data sharing is not applicable to this article as no data sets were generated or analyzed during the current study.
